# Adverse Drug Reactions of Spontaneous Reports in Shanghai Pediatric Population

**DOI:** 10.1371/journal.pone.0089829

**Published:** 2014-02-24

**Authors:** Hui Li, Xiao-Jing Guo, Xiao-Fei Ye, Hong Jiang, Wen-Min Du, Jin-Fang Xu, Xin-Ji Zhang, Jia He

**Affiliations:** 1 Department of Health Statistics, Second Military Medical University, Shanghai, China; 2 Department of politics, Second Military Medical University, Shanghai, China; 3 Adverse Drug Reaction Monitoring Center of Shanghai, Shanghai, China; University of Liverpool, United Kingdom

## Abstract

**Background:**

Knowledge of drug safety in the pediatric population of China is limited. This study was designed to evaluate ADRs in children reported to the spontaneous reporting system (SRS) of Shanghai in 2009.

**Methodology and Principal Findings:**

Crude ADR reports submitted to Shanghai SRS in 2009 for individuals aged from birth to 17 years (including 17 years) were included. Data were analyzed with respect to age, gender, category of ADR (System Organ Class [SOC]), the severity of reports and type of reporter.

**Results:**

A male overrepresentation was observed regarding the total number of reports. The most frequently reported group of drugs were vaccines (42.15%). Skin rash and fever were the commonest symptoms reported in the total pediatric dataset. The proportion of children that suffered from a serious ADR was 2.16% and that for drug related deaths was 0.34%. And we found that the multiple drug exposure experienced a high proportion of serious ADRs compared with the single drug use (χ^2^ = 15.99, P<0.0001). Sixty-five percent of ADRs were for children less than 6 years of age. And more than half of reports were from doctors.

**Conclusions:**

In our study, consumers were more likely to report new ADRs though they appear to contribute a relatively small percentage of total reports. We propose that patients would take an active role in reporting ADRs. More researches are needed in order to achieve better understanding the characteristics of ADRs in pediatric population of China.

## Introduction

Adverse drug reaction (ADRs) are defined as events related to a medication that are noxious, unintended and occur at normal doses used in humans for prophylaxis, diagnosis or therapy of disease, or for modification of physiological function by World Health Organization (WHO). This definition excludes accidental or deliberate excessive dosage or maladministration [1]. ADRs are one of the leading causes of morbidity and mortality in many countries [2], [3]. In the UK a major study of hospital patients found that up to 6.5% of admissions were due to ADRs, and 2.3% of those patients died after admission in hospital [3]. A systematic review have shown that the incidence rates for ADRs causing hospital admission ranged from 0.4% to 10.3% of all children (pooled estimate of 2.9% (2.6%, 3.1%)) and from 0.6% to 16.8% of all children exposed to a drug during hospital stay [4]. In addition, ADRs constitute a major cost factor in public health care. Almost 5% of hospital admissions in the pediatric setting and 10% of hospitalized pediatric patients are presumably due to drug related problems [5], [6], [7]. A large variety of drugs are used in children now [8], although many of them have been marketed without trials regarding the efficacy and safety in this population. Little attention has been paid to the safety of medicines in children [9], [10]. The ADRs resulting from the administration of a drug that has not been test and proven in the pediatric population through proper clinical trials may therefore lead to significant morbidity and death among children [11], [12]. In a meta-analysis of 17 prospective studies performed in the United States and Europe, the overall incidence of ADRs in hospitalized children was estimated to be 9.53% (95% CI 6.8 to 12.6) and in pediatric outpatients 1.46% (95% CI 0.7 to 3.3), and serious reactions accounted for 12% of the total number of ADRs. The contribution of ADRs to the hospitalization of children was estimated to be 2.09% (95% CI 1.02 to 3.77) [7]. Knowledge of drug safety is limited in the pediatric population, especially for off-label drugs. Less than half of those specifically intended for children are operated on the basis of clinical trials which demonstrate specific features of risk-benefit balance in children [13]. Spontaneous reporting of ADRs has been shown to be an important method of increasing such knowledge and the method could be considered particularly important in children since drugs are not routinely test in the pediatric population [14]. A major function of the spontaneous reporting system is early detection of signals of new, rare or serious ADRs. The SRS covers a large number of patients and a wide range of drugs. It is therefore a relatively cost-effective method for drug safety monitoring [15]. However, few studies were conducted to investigate knowledge of drug safety in Chinese children.

The Shanghai ADR SRS is a part of China ADR SRS and one of the major goals of this system is the timely detection of possible new ADRs. There are 12000–16000 ADR reports submitted to the system annually now. Both urban and rural areas in Shanghai are included in this ADR monitoring scheme, covering more than 17,000,000 inhabitants. Pharmaceutical manufactures, healthcare professionals (HCPs), and drug dealers are the main sources of reports. This scheme is a voluntary reporting system without any incentive. The monitoring scheme includes not only prescribed medications, but also over the counter, traditional Chinese remedies, etc. In this study, we demonstrated the characteristics of suspected pediatric ADRs reported to the Adverse Drug Reaction Monitoring Center of Shanghai.

## Methods

### Data Acquisition

The data was downloaded automatically from the system via http://www.adr.gov.cn, which was developed by National Adverse Drug Reaction Monitoring Center of China. We cannot get ADR incidence rates as the true extent of drug use was unknown, so all the data in the manuscript were frequency of reports. Personal information was excluded in our dataset, and individual identify codes in SRS were marked with random numbers. The study was approved by the Ethics Committee of the Second Military Medical University, Shanghai, China. The ethics committee waived the need of informed consent for the study because of its retrospective nature and data were analyzed anonymously.

### Data Coding

The ADR names were coded according to MedDRA (Medical Dictionary for Regulatory Activities, version 3.0.2b) terminology at SOC level. In addition, generic names of drugs were standardized and coded according to the catalogue of generic names for common prescription drugs issued by the Ministry of Health of China in 2007. The website http://app1.sfda.gov.cn/datasearch/face3/dir.html and Chinese Pharmacopoeia were also used as materials for our work [16]. We classified each ADR as a general, serious, new general or new serious reaction based on the Measures for the Reporting and Monitoring of Adverse Drug Reactions definitions [17]. Serious Adverse Drug Reactions means one of the following harmful situations caused by taking drugs: 1. Results in death; 2. Results in cancer, a persistent of significant disability/incapacity; 3. Results in life threatening; 4. A persistent of injury to organ function; 5. Results in hospitalization or prolongs an existing inpatient hospitalization. And new ADRs means adverse reactions that are not recorded and explained on the drug package insert [17]. Age -specific groups were classified into newborn (0–1 month), infant (1–23 months), preschool child (2–5 years), child (6–12 years), and adolescent (13–17 years, including 17 years) [18]. When a suspected ADR is reported more frequently on the combination of two drugs as compared with the situation where these drugs are used in the absence of each other, this association might indicate the existence of a drug-drug interaction (DDI) [19], [20]. By patient reporting we mean a slightly adapted version of the van Grootheest definition: ‘users of drugs (or their parents or cares) reporting suspected ADRs directly to a spontaneous reporting system [21].

### Data Split

ADR report may contain one or more drugs and ADRs. In this study, all drug-event pairs, whether the role of the drug of interest was considered ‘suspect’ or ‘concomitant’, was included and the calculation was based on the counts of drug-event pairs in the analysis.

### Data Analyses

Categorical data were compared by Chi-square test, and ranked data by Cochran-Mantel-Haenszel test. Fisher’s exact test was used when numerators were small number. Odds ratios (ORs) and 95% confidence intervals (CIs) between the rates of new ADRs report in different reporters were calculated using Logistic Regression. Only two-tailed tests were used. A *P*-value of 0.05 or less was considered to be significant and statistical analyses were performed using SAS 9.3 software (SAS Institute, Cary, NC, USA).

## Results

A total of 24292 reports were submitted to Shanghai SRS in 2009, and 3945 reports concerning children 0–17 years (including 17 years) were retrieved from the system. Of the 3945 reports, 97 were deleted from our analyses for the following reasons: drug administration was to the mother (n = 1); out-of-range age (n = 86); no suspected ADR (n = 9); or no suspected drug (n = 1). In total, we examined 3848 reports of suspected pediatric ADRs that were reported to the Adverse Drug Reaction Monitoring Center of Shanghai during the study period.

The 3848 reports included 4430 suspected ADRs, with an average of over 10 reports per day and 1.15 ADRs per child. There were 666 reports, which cited more than one suspected drug (Table 1). The total number of suspected drugs in pediatric reports was 4619 with a mean number of 1.20 drugs per child.

**Table 1:** Number of suspected drugs per ADRs report.

Number of Suspected Drugs Reported (n)	Number of ADR Reports (n)
1	3182
2	583
3	67
4	12
5	3
7	1
Total	3848

### ADRs by Age and Gender

**Figure 1:**
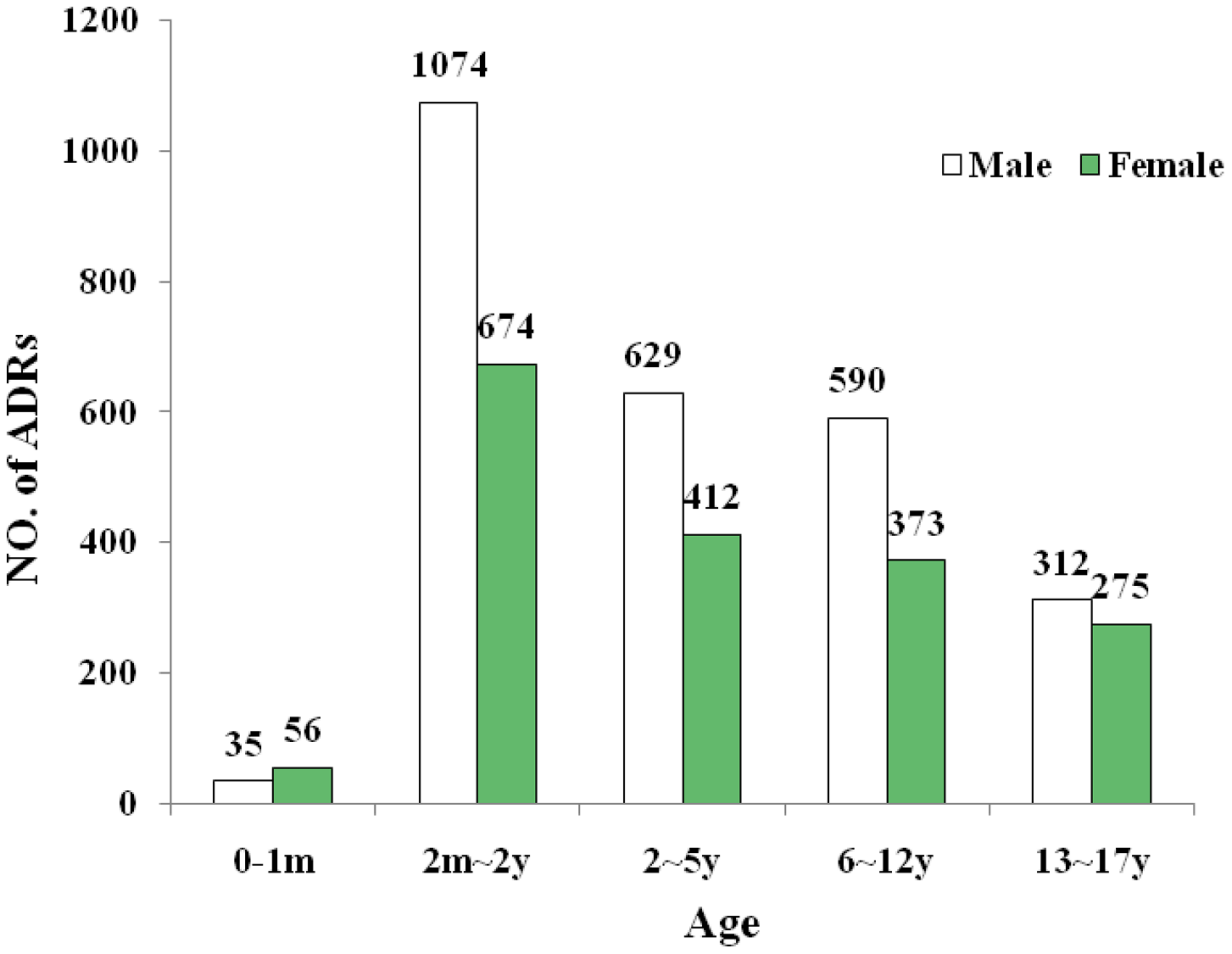
Age- and gender-specific prevalence of ADRs based on the data from Shanghai SRS (2009).

The age- and gender-specific prevalence of ADRs was presented in Fig. 1. In general, a total of 1790 ADRs (40.41%) and 2640 ADRs (59.59%) were reported for female and male patients, respectively. More ADRs were reported for boys than girls except the 0–1 month group. When the data were assessed in terms of age groups, almost two thirds of ADRs were reported for children from birth to 5 years of age (65.01%) and 39.46% concerned children aging 2 months-2 years (Fig. 1). Table 2 showed the number of serious ADRs report of different age groups. The highest proportion (6.58%) of serious reports was reported for newborn (0–1 month). A total of 110 serious ADRs were detected in our analysis with 57 (2.16%) in male patients and 53 (2.96%) in female patients. No statistically significant difference was observed between boys and girls by Fisher exact test (*P* = 0.095, two sided test of proportions).

**Table 2:** Number of ADRs reports by age and severity category.

Age of child	Number of ADR Reports (n)	Number of serious ADR Reports [n (%)]
0–1 month	76	5(6.58)
2 months –23months	1642	25(1.52)
2–5 years	901	21(2.33)
6–12 years	769	15(1.95)
13–17 years	460	17(3.70)
Total	3848	83(2.16)

### ADRs by Vaccines and Non-vaccines

The single most common reaction was exanthema, followed by fever, application site reaction and vomiting (Table 3). Regarding assessment of drugs, the most frequent reports were related to vaccine use (1622 reports, 42.15%). When the non-vaccine related reports were discriminated by excluding children that had been reported to have a suspected ADR of a vaccination, the total number of children, whom an ADR had been related to, were then reduced to 2649. Skin reactions were still most frequently reported. The most commonly reported drugs among serious reports were antibacterials for systemic use (32.82%), nervous system (17.56%) and vaccines (13.74%). When drugs were assessed, cefuroxime (382 reports), azithromycin (340 reports) and cefotiam (130 reports) were the most frequently reported non-vaccine related drugs. When assessing the serious ADRs, the frequency of the non-vaccine related increased to 83.64%, the most frequently reported non-vaccine drugs were ceftriaxone (9 reports), cefuroxime (7 reports) and lamotrigine (7 reports).

**Table 3:** Ten most frequently reported ADRs.

Suspected ADRs (n)	Reaction terms	Percent of all suspected ADRs (%)
1282	Exanthema	28.94
951	Fever	21.47
401	Application site reaction	9.05
195	Vomiting	4.40
165	Urticaria	3.72
161	Pruritus	3.63
136	Nausea	3.07
84	Maculopapule	1.90
79	Diarrhea	1.78
74	Abdominal Pain	1.67

The number of all suspected ADRs was 4430.

### Multiple Drug Exposure and Serious ADRs

Among the total 3848 ADRs reports, there were 3182 single drug use and 666 multiple drug exposure reports (Table 1). Two point forty-eight percent (110/4430) of the adverse drug reactions were reported as serious reactions (48 new serious ADRs). The most frequently reported serious reactions were Anaphylactic Shock (17 reports, 15.45%) followed by exanthema (11 reports, 10.00%). Fifty-five (1.73%) and 28 (4.20%) reports in single drug use and multiple drug exposure, respectively, were registered as serious. It seemed that multiple drug exposure experienced a high proportion of serious ADRs compared with the single drug use (χ^2^ = 15.99, *P*<0.0001, two sided test of proportions).

### Outcomes of ADRs Reports

Of all 3848 children, 1176 (30.56%), 2655(69.00%), 4(0.10%), 13(0.34%) children were reported to be cured, getting better, recovering with sequelae, and death, respectively (Table 4). Vaccine accounted for 50% of these cases, and others were central nervous system agents. The fraction of non-vaccine related reports with total recovery of the patients was 30.10% (670) and 69.45% (1546) of children were getting better. Two children had recovered with sequelae of non-vaccine related reports. There were 13 drug related deaths, with 8 of non-vaccine and 5 vaccine related reports (Table 5).

**Table 4:** Outcomes resulting from ADR Reports.

Outcome	Number of ADR Reports [n (%)]
Cure	1176(30.56)
Getting better	2655(69.00)
Recovering with sequelae	4(0.10)
Death	13(0.34)
Total	3848(100.00)

**Table 5:** Reported adverse drug reactions (ADRs) in children leading to death.

Case	Sex	Age (year)	Suspected drugs	ADR(s) reported
1	Male	0.33	Pediatric Pseudoephedrine Hydrochloride andDextromethorphan Hydrochloride Drops	Sudden death
2	Male	0.35	Bacillus Calmette-Guerin Vaccine	Tubercle bacillus infection
3	Female	0.40	Diphtheria-Tetanus-Pertussis Vaccine	Sudden death
4	Female	0.40	Diphtheria-Tetanus-Pertussis Vaccine	Anaphylactic Shock, Sudden death
5	Male	0.41	Ambroxol, Amoxicillin, Benzylpenicillin, Terbutaline Sulfate	Diarrhea, Crying
6	Female	0.58	Diphtheria-Tetanus-Pertussis Vaccine	Sudden death
7	Male	0.92	Cefuroxime, Ambroxol, Cefradine, Terbutaline	Sudden death
8	Male	0.99	Ceftriaxone	Hand-foot-and-mouth disease aggravated
9	Female	1.73	B-Haemophilus influenza Conjugate Vaccines	Encephalitis, Interstitial pneumonia
10	Male	2.14	Cefotaxime, Glucose	Convulsion, Sudden death
11	Female	4.53	Cefuroxime	Anaphylactic Shock
12	Female	12.44	Ceftriaxone, Glucose	Dyspnea, Sudden death
13	Female	16.23	Mycophemolate Mofeil, Prednisone	Pneumonia

### ADRs by Type of Reporter and Severity

Analysis of the ADR reports for children received in 2009 of spontaneous reporting system (SRS) of Shanghai showed that 9.68% (429) of the suspected ADRs were described as new to the Agency, of which 381 ADRs were general and 48 ADRs were serious. There were 52.03% (2002 reports), 24.27% (934 reports) and 15.46% (595 reports) of reports from physicians, pharmacists and other HCPs, respectively. For the total 3848 reports, Spontaneous reports from consumers (like patients themselves or their parents) appear to contribute a relatively small percentage of total reports (2.52%, 97 reports). Nearly 5.72% (220) of reporters did not give their occupations. The difference on distributions of severity between different reporters was not statistically significant (χ^2^_CMH = _3.09, *P* = 0.377). However, it was concluded that the sources of reports might have a difference between new and traditional ADRs (χ^2^_CMH_ = 22.45, *P*<0.0001). When compared with reports from other HCPs, consumers were more likely to report new ADRs (OR = 5.06, 95% CI 1.84 to 13.94, Table 6).

**Table 6:** The Logistic regression results of different reporters (new or traditional ADRs).

Reporter	Wald χ^2^	*P*	OR (95%CI)
Physicians vs. consumers	5.88	0.0153	1.13(0.51,2.49)
Pharmacists vs. consumers	2.96	0.0855	1.22(0.54,2.76)
Other healthcare professionalsvs. consumers	17.27	<0.0001	5.06(1.84,13.94)

## Discussion

In our study, more than 50% of the ADRs were reported for children from birth to 5 years of age and almost 40% concerned children between 2 months and 2 years of age. Similar findings were observed in other studies [22], [23], [24]. Several reasons might contribute to the higher reporting rates in young children. Firstly, young children are more closely monitored by physicians and parents. Secondly, a large number of ADRs reported in this age group may also be due to the widely use of off-label and unlicensed drugs. Thirdly, Children under 5 were the most common age group for vaccination. The ADR rate causes by vaccine is much higher than other drugs, and this may be related to the types and number of vaccination being used in China, as the types of routine immunization vaccines in China reach up to 15 kinds, which is much higher than 7 kinds in India and Vietnam, 9 kinds in Thailand and 11 kinds in America, and most of the vaccines in China are attenuated live vaccines, which may bring greater potential safety hazard. In addition, previous studies suggested an increased risk of ADRs for drugs used off-label [25], [26], [27]. This was an important issue regarding children’s health risk. Some studies in adults demonstrated that female patients were more prone to develop ADRs than male patients whereas other studies did not [28], [29]. However, a recent pediatric study published in 2011 found that a high proportion of ADR reports among children were for boys [30]. For our research, there seems to be an overrepresentation of male patients in the ADR reports except the 0–1 month group, which is consistent with Star’s finding [30]. This may be explained by more attention being paid to ADRs in males than females in some parts of China or it is an indication that male patients truly suffer more often from ADRs than female ones. Further investigations are needed to explain this finding.

In the present survey, skin reactions were the most frequently reported ADRs, regardless of including or excluding of vaccine-related reports. And this is consistent with previous findings [23], [31]. Antibiotics were the most frequently reported pharmacological group of drugs in previous studies in contrast to our study where vaccines were most commonly reported [31], [32], [33]. This difference might be due to the occurrence of Type A H1N1 influenza in China this year, and HCPs or parents paid close attention to children who got flu vaccine to prevent the influenza. Our study showed that the majority of the children recovered without sequelae and about 1.04% recovered with sequelae. Vaccines were associated with the highest number of Children that recovered with sequelae, followed by central nervous system agents.

With the seemingly constant flow of new therapeutic agents and new treatment indications for existing medications, polypharmacy is increasingly common [34], [35]. Drug-drug interactions (DDI) occur when two or more drugs are taken in combination and one drug influences the effects of another drug. This may subsequently cause a change in the pharmacodynamic or pharmacokinetic parameters which may lead to lack of efficacy, or to an increase in the number of reported adverse drug reactions. The association between multiple drug exposure and the incidence of ADRs has been studied, consistently showing an exponentially increased risk with the increase of the number of drugs taken [36], [37]. When assessing the severity of the reported ADRs, our study confirmed that multiple drug exposure experienced a high proportion compared with the single drug use. This finding indicate that in order to minimize the risk of serious ADRs, HCPs should pay particular attention to children who are prescribed two drugs or more.

The majority of ADRs in children were reported by physicians, and equal shares of serious ADRs were reported by physicians, pharmacists, other HCPs and consumers. This was different with studies of other countries [22], [33]. Previous founding suggested that patients’ reports were more likely to be serious ADRs than health professional reports [38]. In our study, there were just a small proportion of reports from patients themselves or their parents, but they might report more new ADRs. The importance of patient reporting in not only contributing to ‘signal generation’ but also providing data on ‘adverse changes in the quality of life which can be very important, real and distressing to the medicine user yet are unlikely to be clear to a prescriber’ was also highlighted [39]. Patient reporting of suspected ADRs in Shanghai should be encouraged and their reports should be taken as seriously as reports from other sources.

There is so little known of ADRs in the Chinese population, especially for the children. Our study analyzed information reported to Shanghai ADR database on ADRs in pediatric population, and in conclusion, a male overrepresentation was observed regarding the total number of reports, most ADRs were for children less than 6 years of age, the multiple drug exposure experienced a high proportion of serious ADRs, and consumers just contributed a limited number of ADR reports. There were also several limitations of this study. We just evaluated ADRs in children reported to the SRS of Shanghai in 2009, in order to achieve better understanding the characteristics of ADRs in pediatric population more researches are needed. Substantial underreporting of ADRs is a well-known phenomenon, which makes it difficult to estimate the ADR incidence for pediatric patients. In this study, the results suggest that 2.16% of all ADRs were severe, and 0.34% were fatal. However, the true proportion is likely to be much lower than this because severe reactions are much more likely to be reported. We recommend more prospective investigations in outpatient and inpatient settings to better estimate the type and incidence of ADRs in Chinese children.
